# UPLC-Tandem Mass Spectrometry Method for Simultaneous Determination of Fluoxetine, Risperidone, and Its Active Metabolite 9-Hydroxyrisperidone in Plasma: Application to Pharmacokinetics Study in Rats

**DOI:** 10.1155/2017/5187084

**Published:** 2017-06-01

**Authors:** Essam Ezzeldin, Nisreen F. Abo-Talib, Marwa H. Tammam

**Affiliations:** ^1^Pharmaceutical Chemistry Department and Drug Bioavailability Laboratory, College of Pharmacy, King Saud University, P.O. Box 2457, Riyadh 11451, Saudi Arabia; ^2^Drug Bioavailability Center, National Organization for Drug Control and Research, P.O. Box 29, Cairo, Egypt

## Abstract

Risperidone (RIS) is used as an antipsychotic drug alone or with other drugs, like fluoxetine (FLX). A simple method was developed and validated for the determination of both RIS and its metabolite 9-hydroxyrisperidone (9-OH-RIS), FLX, and olanzapine (OLA) as an internal standard in rat's plasma using UPLC-MS/MS. FLX, RIS, 9-OH-RIS, and OLA were purified using acetonitrile as a protein precipitating agent. Separation was performed on an ACQUITY™ “UPLC BEH™” C18 column (50 mm × 2.1 mm i.d., 1.7 *μ*m; Waters Corp., USA). The ranges of the calibration curves were 1.0–1000.0 ng/mL for FLX and 0.2–1000.0 ng/mL for RIS and 9-OH-RIS. Linearity, recovery, precision, and stability were within the acceptable range. This method is rapid, fast, and precise for the determination of RIS and FLX in plasma and is applicable in pharmacokinetic studies.

## 1. Introduction

**Figure 1:**
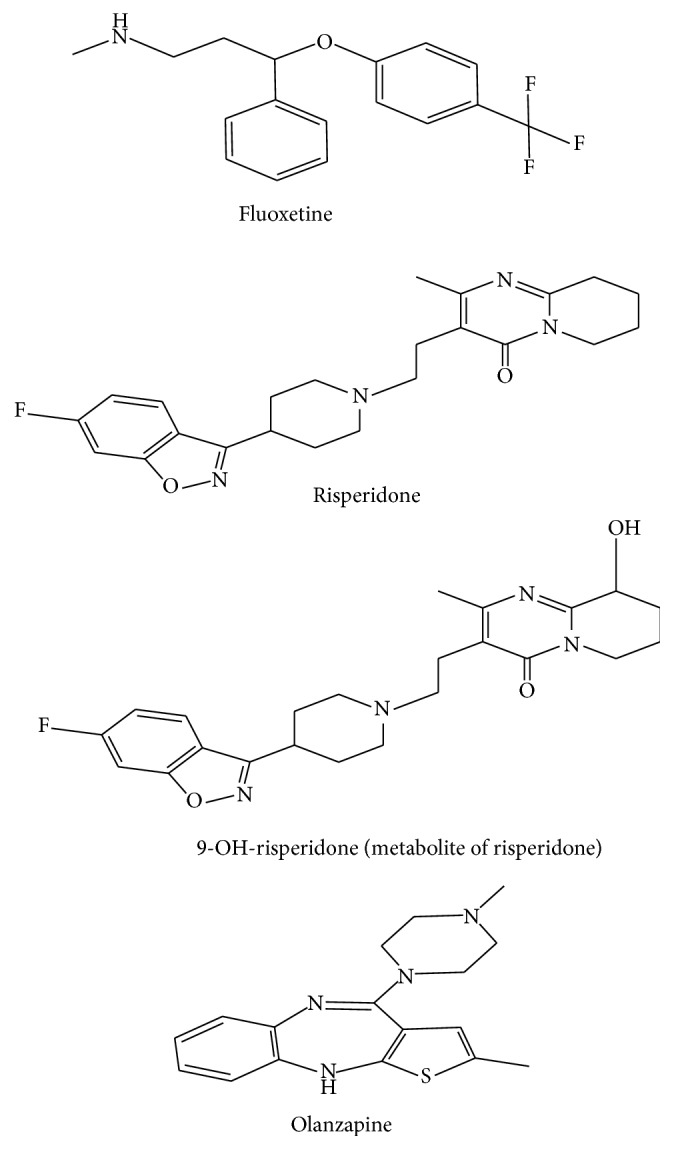
Chemical structure of fluoxetine, risperidone, 9-OH-risperidone, and olanzapine.

Fluoxetine (FLX), which has the full formula (3*RS*)-N-methyl-3-phenyl-3-[4-(trifluoromethyl)phenoxy]propan-1-amine hydrochloride (Figure 1), is a selective serotonin reuptake inhibitor antidepressant drug [1] with comparable effects to those of tricyclic antidepressants [2]. Maximum FLX plasma concentration is reached 6–8 h after oral administration. Pharmacokinetic studies have shown that FLX has a long half-life, causing it to be administered on a weekly basis [3]. Furthermore, FLX has fewer cardiovascular and anticholinergic side effects than comparable drugs [2].

Risperidone (RIS), which has the full formula (3-[2-[4-(6-fluoro-1,2-benzisoxazol-3-yl)-1-piperidinyl]ethyl]-6,7,8,9-tetrahydro-2-methyl-4H-pyrido[1,2-a]pyrimidin-4-one) (Figure 1), is a benzisoxazole antipsychotic agent used to treat schizophrenia and other psychoses. It is more effective and produces fewer side effects than typical antipsychotics [4]. Following oral administration, RIS is rapidly absorbed. The drug is metabolized mainly by the liver, with less than 1% being excreted unchanged in the feces. In order of preference, the major metabolic pathways include 9-hydroxylation,* N*-dealkylation, and 7-hydroxylation [5]. 9-Hydroxyrisperidone (9-OH-RIS) is the principal metabolite of RIS and is the only one that has the same therapeutic effects [6].

Several methods developed for determining FLX in plasma, serum, and pharmaceutical preparations have been published. These include titrimetric method [7], nuclear magnetic resonance (NMR) [8], potentiometry [9], thin-layer chromatography (TLC) [10], liquid chromatography (LC) [11–14], gas chromatography (GC) [15, 16], and electrophoresis [17, 18].

RIS and 9-OH-RIS are most commonly determined by high-performance liquid chromatography (HPLC) using ultraviolet detection (UVD) [19–22] or electrochemical detection (ED) [23–25], respectively. Recently, RIS and 9-OH-RIS have been determined by HPLC combined with mass spectrometry (MS) [26–29].

Currently prescribed antidepressant drugs are only partially effective, and considerable research has been conducted for developing more efficient pharmacotherapies. One option is combined treatments using first-line antidepressants and other drugs with different modes of action, for example,* N*-methyl-D-aspartate (NMDA) receptor antagonists [30], cyclooxygenase inhibitors [31], and atypical antipsychotics [32]. RIS is one such atypical antipsychotic, and its use in treating depressive disorders has been reported [33, 34].

Consequently, monitoring the level of both FLX and RIS in biological fluids is essential. However, no methods for the determination of FLX and RIS simultaneously have been published. Therefore, this study aims at developing a method for the selective determination of RIS, 9-OH-RIS, and FLX using UPLC-MS/MS.

## 2. Experimental

### 2.1. Chemicals and Reagents

FLX (99.4%), RIS (99.6%), and 9-OH-RIS (99.5%) standards were purchased from Sigma-Aldrich, USA. Olanzapine (OLA) was kindly supplied by Janssen-Cilag, Belgium, and used as an internal standard (IS). Acetonitrile and methanol (HPLC grade) were purchased from Alpha Chemicals, Egypt. Formic acid and ammonium acetate were purchased from Romil Chemicals, England. Deionized water was obtained from a Milli-Q water purification system (Millipore, France).

### 2.2. Instrumentation

Chromatography was performed on an ACQUITY UPLC system coupled with a triple-quadrupole tandem mass spectrometer (Waters Corp., Milford, MA, USA). Separation of the analytes was performed on an ACQUITY UPLC BEH C18 column (50 mm × 2.1 mm i.d., 1.7 *μ*m; Waters Corp., USA) maintained at 40°C. The mobile phase was 80 : 20 (v/v) mixture of 0.1% formic acid in acetonitrile and 0.1% formic acid in 0.25 M ammonium acetate buffer at a flow rate of 0.6 mL/min. The injection volume was 5 *μ*L in partial-loop mode, and the temperature of the autosampler was kept at 10°C. Multiple reaction monitoring (MRM) in electrospray positive ion mode was used for detection and quantitation of all analytes. The MRM transitions selected and mass optimization parameters are summarized in Table 1. “Mass Lynx” software (Version 4.1) was used for evaluation of peak areas.

### 2.3. Animals

All animal experiments were carried out under animal use regulations. Wistar rats (200−250 g) were obtained from the Laboratory Animal Center (NODCAR, Egypt). Animals were acclimated for at least five days and fasted overnight before the experiments.

### 2.4. Preparation of Standard Solutions

Standard 100.0 *µ*g/mL solutions of FLX, RIS, 9-OH-RIS, and OLA were prepared in methanol. All solutions were stored at 4°C and brought to room temperature before use, and they were used for 15 days from the date of preparation.

### 2.5. Calibration Curves and Quality Control Samples

To construct plasma calibration standards, appropriate amounts of the diluted stock FLX, RIS, and 9-OH-RIS methanol solutions were added to blank plasma to yield final concentrations of 1.0, 5.0, 10.0, 100.0, 500.0, and 1000.0 ng/mL for FLX and 0.2, 0.5, 10.0, 100.0, 500.0, and 1000.0 ng/mL for RIS and 9-OH-RIS. Quality control (QC) samples, denoted as LQC, MQC, and HQC, containing 1.0, 100.0, and 1000.0 ng/mL of FLX, RIS, and 9-OH-RIS, respectively, were prepared. Samples were kept at −80°C.

### 2.6. Sample Preparation

After thawing the samples at room temperature, they were mixed with a vortex mixer prior to sample preparation to ensure complete mixing of the contents. A 100 *μ*L plasma sample was pipetted into a 10 mL glass test tube. Then, 10 *μ*L of IS (12.0 *µ*g/mL) was added, and the sample was mixed with vortex for 30 s. Subsequently, 300 *μ*L of acetonitrile was added for protein precipitation and the mixture was shaken by vortex and centrifuged for 10 min at 4500 rpm at 4°C. Then, the supernatant was transferred to a clean vial, and 5 *μ*L was injected into the UPLC-MS/MS apparatus for analysis.

### 2.7. Method Validation

UPLC-MS/MS assay validation was performed according to the US FDA guidelines [35]. The selectivity of the method was investigated by comparing detector response at the retention times of plasma samples spiked at the lower limit of quantification (LLOQ) (1.0 ng/mL for FLX and 0.2 ng/mL for RIS and 9-OH-RIS and at 1200.0 ng/mL for the IS) with those from free-drug plasma.

The linearity of the method was determined by analysis of twelve standard calibration curves with six different concentrations ranging from 1.0 to 1000.0 ng/mL for FLX and from 0.2 to 1000.0 ng/mL for RIS and 9-OH-RIS. The correlation coefficient (*r*^2^) was >0.999 for all the calibration curves. The ratio of peak-area response of the analyte to IS was used for regression analysis. The concentration of the drug in rats samples was calculated from the calibration curve (*y* = *bx* + *a*) and the regression coefficient was calculated. The LLOQ is the lowest concentration of the analyte on the calibration curve which is 1.0 ng/mL for FLX and 0.2 ng/mL for RIS and 9-OH-RIS.

Assay precision is expressed as percentage of variation (% CV) while the deviation of the concentration was found from the nominal one expressed as the accuracy.

Precision and accuracy during intraday and interday of the method were measured by injection of three QC samples (LQC, MQC, and HQC) in six replicates on the same day and on successive days, respectively. Deviation values for these parameters should be within 20% for the LLOQ and 15% for the QCs above the LLOQ.

The recovery of an analytical method is defined as a comparison between detector response for the concentration of the authentic sample and the response of the detector for the same concentration added and extracted from a biological matrix. The extraction recoveries of FLX, RIS, and 9-OH-RIS were determined at three concentration levels each.

The stability of the analytes in rat plasma during sample storage as well as during processing conditions was assessed by analyzing the LQC, MQC, and HQC with six replicates. Short-term stability indicated acceptable stability behavior during the experimental conditions of the regular runs at ambient temperature for 6 h. Freeze-thaw plasma stability was checked over three freeze-thaw cycles after storage in ultradeep freezer. The long-term stability was determined after storage at −80°C for 6 weeks. Postpreparation stability was measured by reanalyzing the extracted plasma samples kept under the autosampler conditions for 24 h.

### 2.8. Application of the Method in a Clinical Pharmacokinetic Study

The present method was fruitfully applied for determinations of FLX, RIS, and 9-OH-RIS levels in rat plasma samples. A pharmacokinetic study was conducted using six male Wistar rats (200−250 g). After overnight fasting, the rats received simultaneous oral doses of FLX (10 mg/kg) and RIS (0.3 mg/kg). Blood samples (0.5 ml) were collected at different time intervals. Plasma samples were centrifuged at 4000 rpm and the separated plasma samples were stored in an ultradeep freezer until analysis. Different pharmacokinetic parameters were estimated for each rat.

## 3. Results and Discussion

In biological matrices, quantification of drugs by LC-MS/MS is widespread due to the high sensitivity and selectivity of this technique. Such sensitivity is fundamental to establish a method capable of quantifying FLX, RIS, and 9-OH-RIS at a level down to 1.0 for FLX and 0.2 ng/mL for RIS and 9-OH-RIS. The ingrained selectivity of MS/MS detection was expected to be helpful in developing a selective and sensitive method. Furthermore, this method would be suitable for efficient analysis of a large number of plasma samples for pharmacokinetic, bioavailability, and bioequivalence studies of FLX and RIS.

There is no reported method for the determination of FLX and RIS and 9-OH-RIS in plasma simultaneously; therefore, the aim of this study was to develop and validate a simple, fast, and specific UPLC-MS/MS assay method for simultaneous extraction, separation, and quantification of the cited drugs. To achieve this goal, different selections were estimated during the development of the method to optimize detection parameters, chromatographic separation, and sample extraction.

**Figure 2:**
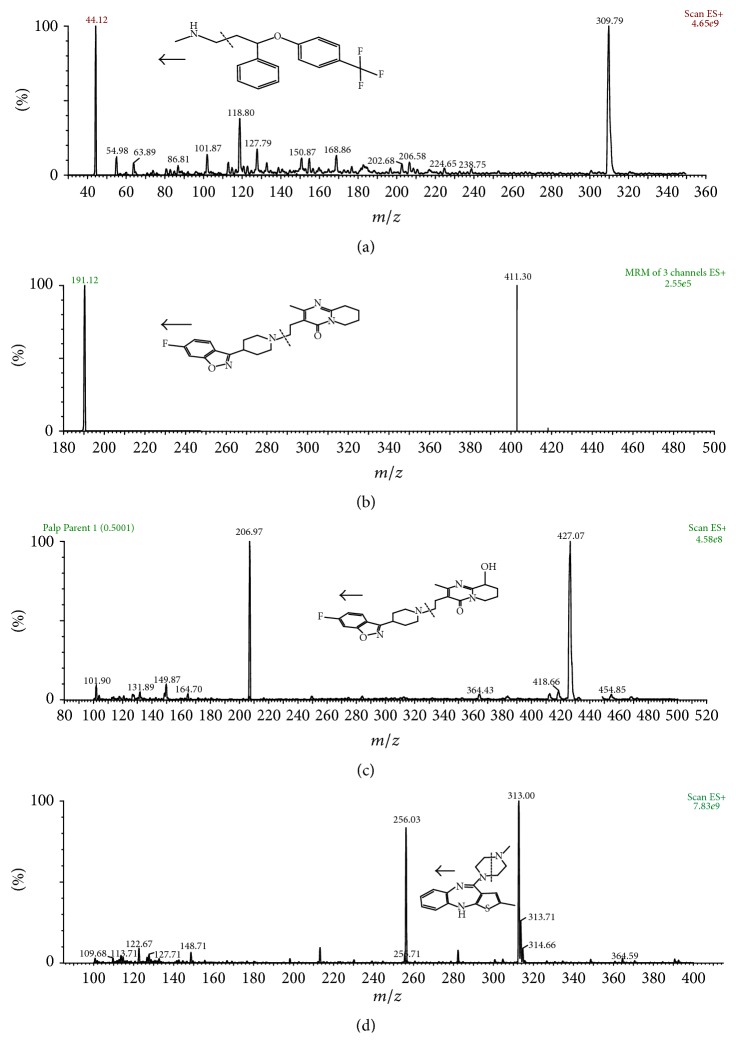
Full scan positive ion mass spectra and the proposed fragmentation of (a) FLX, (b) RIS, (c) 9-OH-RIS, and (d) OLA (IS).

LC-multiple reaction monitoring (MRM) is a great technique as it provides the sensitivity and selectivity required for accurate analysis. Thus, the MRM technique was chosen for our method. Electrospray ionization (ESI) was employed in order to obtain a better response from the analytes. The best signals were achieved using ESI-positive ion mode. The product ion mass spectra for FLX, RIS, 9-OH-RIS, and OLA present a high abundance of fragment ions of* m/z* 44.18, 191.12, 206.97, and 256.03, respectively (Figure 2).

### 3.1. Method Development

The constituents of the mobile phase were changed several times to achieve a chromatogram with symmetric peak and good resolution for the analytes and IS. A mixture of 0.1% formic acid in acetonitrile and 0.1% formic acid in 0.25 M ammonium acetate buffer (80 : 20, v/v) with a flow rate of 0.6 mL/min achieves this purpose and permits a run time of 2.0 min. Endogenous substances in the plasma may affect the column, MS system, and analytes and the IS, which leads to ion suppression. The advantage of protein precipitation is that it helps in preparing a clean sample and consequently avoids this suppression effect in UPLC-MS/MS analysis.

### 3.2. Method Performance and Validation

**Figure 3:**
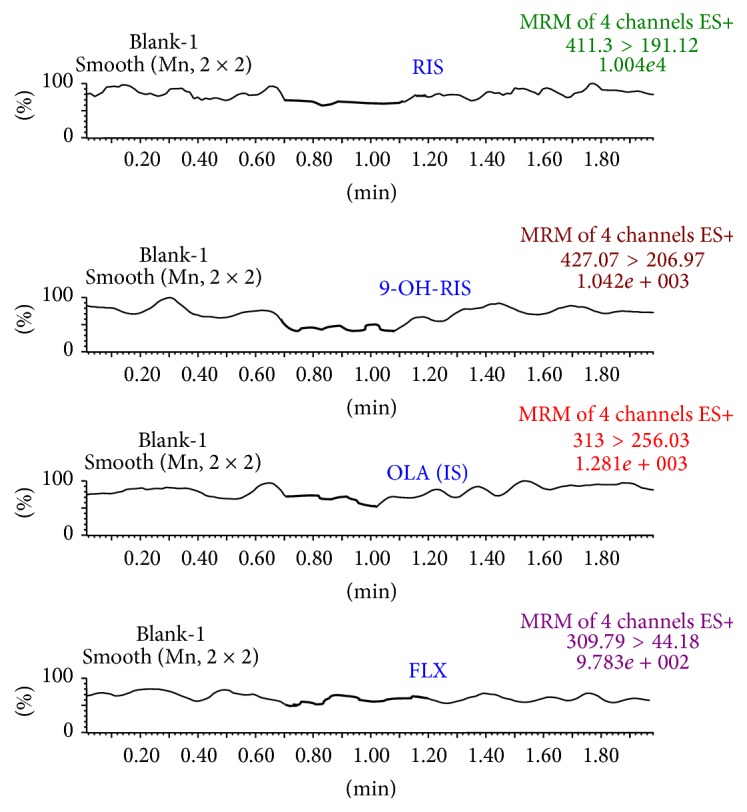
MRM chromatogram for FLX, RIS, and 9-OH-RIS and the IS (OLA) resulting from analysis of blank plasma.

**Figure 4:**
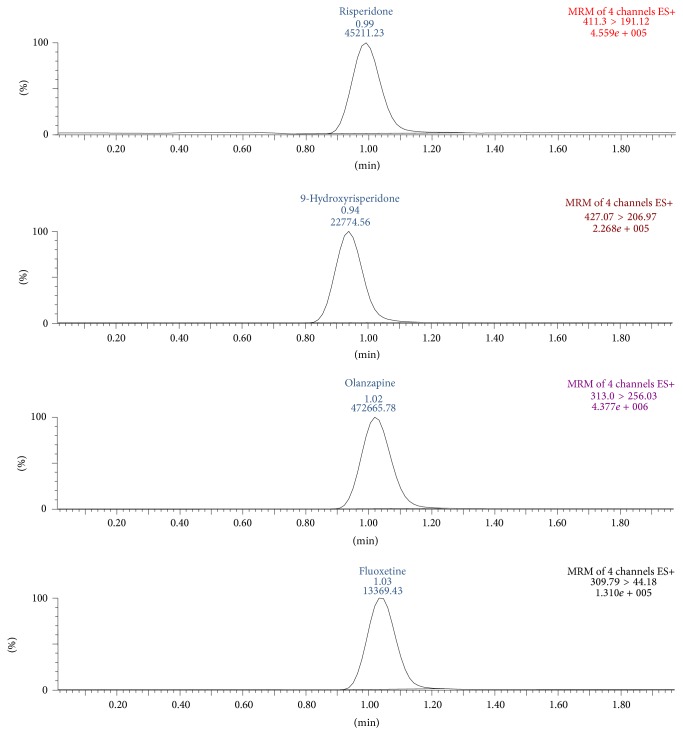
MRM chromatogram of plasma spiked with 100.0 ng/mL of FLX, RIS, and 9-OH-RIS and 1200.0 ng/mL of the IS (OLA).

A representative chromatogram obtained from blank plasma is shown in Figure 3. The MRM chromatograms obtained from spiked plasma samples are shown in Figure 4. No endogenous compounds appear at the retention times of FLX, RIS, 9-OH-RIS, or the IS to interfere with their peaks. Moreover, the base line is relatively free from drift.

**Figure 5:**
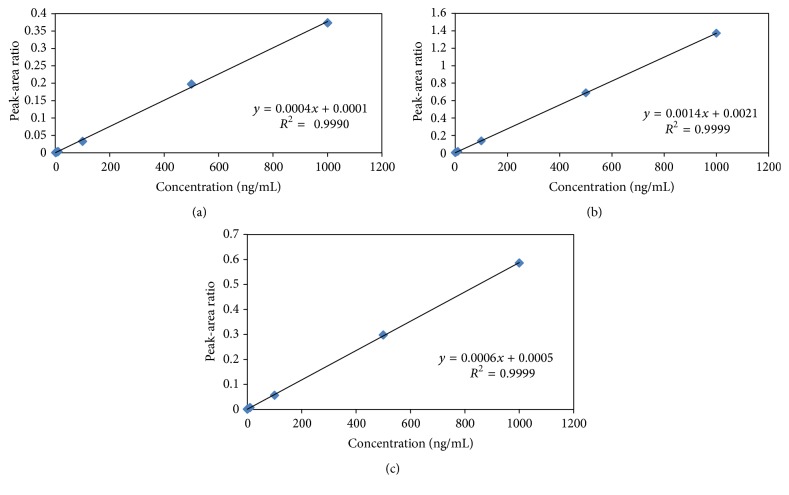
Standard calibration curves of (a) FLX, (b) RIS, and (c) 9-OH-RIS.

The linearity of the method was determined using coefficient of variation of the standard. Calibration curves were obtained by plotting the peak-area ratio (drug/IS) against the concentration of the analyte in the plasma. The linearity of the calibration curves (*n* = 12) was verified from 1.0 to 1000.0 ng/mL for FLX and from 0.2 to 1000.0 ng/mL for RIS and 9-OH-RIS (Figure 5).

The LLOQ is defined as the lowest concentration of an analyte that can be measured accurately under the mentioned experimental condition and meet the acceptable criteria (precision < 20% and an accuracy between 80% and 120%). The LLOQ is 1.0 ng/mL for FLX and 0.2 ng/mL for RIS and 9-OH-RIS. Results of precisions (% CV) and accuracy for the intra- and interday analysis of FLX, RIS, and 9-OH-RIS in plasma are presented in Table 2.

The extraction recovery determined for FLX, RIS, and 9-OH-RIS is shown to be consistent, accurate, and reproducible. The average recovery was 90.54%, 96.41%, and 84.34% for FLX, RIS, and 9-OH-RIS, respectively, which is acceptable for the routine measurement of these analytes (Table 3).

Table 4 summarizes stability data for FLX, RIS, and 9-OH-RIS during analysis. All the results indicate reliable stability behavior during these tests. Therefore, there is no stability-related problem during the routine analysis of samples for the bioavailability study.

**Figure 6:**
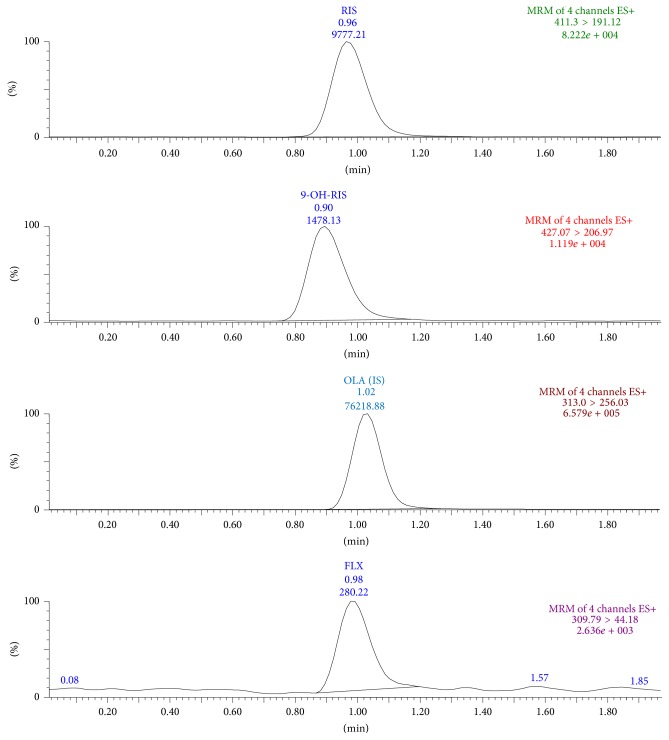
MRM chromatogram of plasma sample from a rat at 1 hr after administration of oral dosing of 10 mg/kg of FLX and 0.3 mg/kg of RIS.

**Figure 7:**
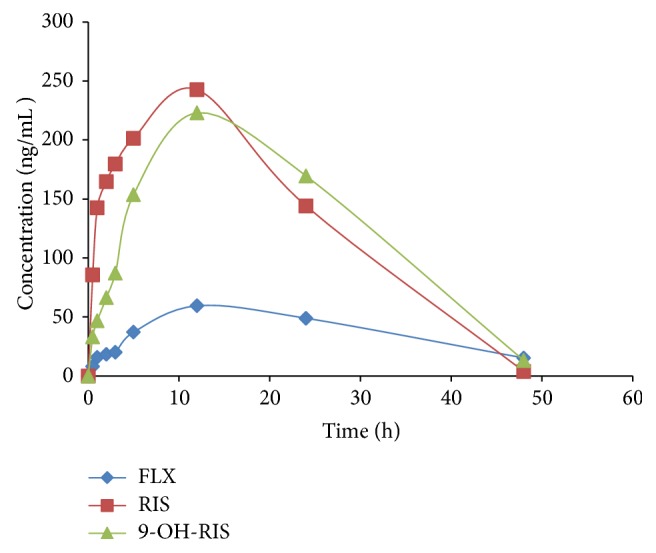
Mean plasma concentration-time profiles after a single oral dose of 10 mg/kg of FLX and 0.3 mg/kg of RIS.

Six male rats received a single oral dose of 10 mg/kg of FLX and 0.3 mg/kg of RIS concurrently and plasma drug levels were determined. The chromatogram of a plasma sample extracted from a rat at 1 h is shown in Figure 6. The concentration-time profiles of FLX, RIS, and 9-OH-RIS are shown in Figure 7. The pharmacokinetic parameters are listed in Table 5.

## 4. Conclusion

In this study, a consistent, selective, and specific and fully validated UHPLC-MS/MS method was developed for the determination of FLX, RIS, and 9-OH-RIS in rat plasma. This method was successfully applied in pharmacokinetic studies in rats. Shorter run time as well as simplicity of sample preparation and wide range of calibration curves allows this method to be applied in monitoring and clinical studies.

## Figures and Tables

**Table 1:** Mass optimization parameters for FLX, RIS, 9-OH-RIS, and OLA.

Parameters	FLX	RIS	9-OH-RIS	OLA
	Source-dependent parameters			
SRM transition (*m*/*z*)				
Parent	309.79	411.3	427.07	313.0
Daughter	44.18	191.12	206.97	256.03
Collision energy (eV)	15	45	45	40
Cone voltages	20	30	25	20

	Compound-dependent parameters			

Collision gas	Argon with a flow rate of 0.1 ML/min			
Desolvating gas	Nitrogen with flow rate of 600 L/h			
Desolvating temperature	350			
Source temperature	150			
Capillary voltage	4 kV			

**Table 2:** Intraday and interday precision and accuracy of FLX, RIS, and 9- OH-RIS in rat plasma.

Nominal conc. (ng/ml)	Intraday reproducibility			Interday reproducibility		
	Mean ± SD	Precision (% CV)	Accuracy (%)	Mean ± SD	Precision (% CV)	Accuracy (%)
FLX						
1.0	1.13 ± 0.24	17.94	113.98	1.18 ± 0.19	16.09	118.16
100.0	88.05 ± 10.06	13.45	88.05	82.59 ± 10.79	13.06	82.59
1000.0	971.16 ± 22.92	2.46	97.12	977.51 ± 29.49	3.02	97.75

RIS						
0.2	0.22 ± 0.02	11.16	109.5	0.21 ± 0.03	15.2	103.64
100.0	92.05 ± 11.77	12.79	92.05	96.44 ± 13.52	14.02	96.44
1000.0	1031.92 ± 3907	3.79	103.19	1028.48 ± 32.57	3.17	102.8

9-OH-RIS						
0.2	0.24 ± 0.02	9.46	120.7	0.22 ± 0.04	19.36	109.6
100.0	90.47 ± 3.97	4.39	90.47	92.81 ± 5.59	6.03	92.81
1000.0	981.79 ± 34.05	3.47	98.18	962.03 ± 31.87	3.31	96.2

**Table 3:** Recovery data of FLX, RIS, and 9-OH-RIS (three QC samples each) in rat plasma (mean ± SD).

Analyte	Nominal conc.	Recovery (%)
FLX	1.0	92.99
	100.0	87.15
	1000.0	91.48
Average		90.54

RIS	0.2	95.88
	100.0	93.79
	1000.0	99.55
Average		96.41

9-OH-RIS	0.2	91.59
	100.0	78.20
	1000.0	83.21
Average		84.34

**Table 4:** Data showing the stability of FLX, RIS, and 9-OH-RIS in human rat plasma at different QC levels (*n* = 6).

Parameters	Stability					
	1.0 ng/mL		100.0 ng/mL		500.0 ng/mL	
	%	CV%	%	CV%	%	CV%
*FLX *						
Bench top (6 hrs)	99.87	4.75	99.87	4.75	101.55	6.93
Freeze-thaw (3 cycles)	97.99	0.20	97.99	0.20	102.61	5.24
6 weeks at −80°C	93.50	6.43	93.50	6.43	106.82	4.76
In autosampler (24 hrs)	98.04	1.58	98.04	1.58	97.95	2.58

*RIS *						
Bench top (6 hrs)	101.15	10.59	101.15	10.59	98.57	4.15
Freeze-thaw (3 cycles)	95.33	6.61	95.33	6.61	98.75	7.53
6 weeks at −80°C	93.99	3.31	93.99	3.31	102.60	4.00
In autosampler (24 hrs)	97.74	2.51	97.74	2.51	98.23	1.43

*9-OH-RIS *						
Bench top (6 hrs)	99.68	3.94	99.68	3.94	109.23	6.05
Freeze-thaw (3 cycles)	97.85	10.15	97.85	10.15	112.61	4.12
6 weeks at −80°C	103.58	6.85	103.58	6.85	110.14	7.71
In autosampler (24 hrs)	99.12	4.11	99.12	4.11	102.10	9.41

**Table 5:** Pharmacokinetic parameters of FLX, RIS, and 9-OH-RIS after oral concurrent administration of FLX and RIS to rats.

Parameters	FLX	RIS	9-OH-RIS
*C*_max_ (ng/ml)	59.50 ± 6.66	234.52 ± 11.43	222.89 ± 15.86
AUC_0–48_ (ng·h/ml)	1861.56 ± 25.63	6434.70 ± 641.76	6360.01 ± 246.78
AUC_0–inf_ (ng·h/ml)	2293.23 ± 196.09	6472.87 ± 652.21	6718.81 ± 184.35
*K*_el_ (h)	0.243 ± 0.29	0.1205 ± 0.009	0.055 ± 0.005

## References

[1] Soni H., Sweetman S. C. (2002). *Martindale—The Complete Drug Reference*.

[2] Wernicke J. F. (1985). The side effect profile and safety of fluoxetine. *Journal of Clinical Psychiatry*.

[3] Pooterand W. Z., Hollister L. E., Katzung B. G. (2004). Antidepressant agents. *Basic & Clinical Pharmacology*.

[4] Meltzer H. Y., Davis K. L., Charney Coyle J. T., Nemeroff C. (2002). Mechanism of action of atypical antipsychotic drugs. *Neuropsy Chopharmacology*.

[5] Huang M.-L., Van Peer A., Woestenborghs R. (1993). Pharmacokinetics of the novel antipsychotic agent risperidone and the prolactin response in healthy subjects. *Clinical Pharmacology and Therapeutics*.

[6] Heykants J., Huang M. L., Mannens G. (1994). The pharmacokinetics of risperidone in humans: a summary. *Journal of Clinical Psychiatry*.

[7] Bueno F., Bergold A. M., Fröehlich P. E. (1999). Assay of fluoxetine hydrochloride by titrimetric and HPLC methods. *Bollettino Chimico Farmaceutico*.

[8] Shamsipur M., Dastjerdi L. S., Haghgoo S., Armspach D., Matt D., Aboul-Enein H. Y. (2007). Chiral selectors for enantioresolution and quantitation of the antidepressant drug fluoxetine in pharmaceutical formulations by 19F NMR spectroscopic method. *Analytica Chimica Acta*.

[9] Atta-Politou J., Skopelitis I., Apatsidis I., Koupparis M. (2000). In vitro study on fluoxetine adsorption onto charcoal using potentiometry. *European Journal of Pharmaceutical Sciences*.

[10] Shah C. R., Shah N. J., Suhagia B. N., Patel N. M. (2007). Simultaneous assay of olanzapine and fluoxetine in tablets by column high-performance liquid chromatography and high-performance thin-layer chromatography. *Journal of AOAC International*.

[11] Yilmaz N., Özkan Y., Özkan S. A., Biryol I., Aboul-Enein H. Y. (2000). High performance liquid chromatographic assay and drug dissolution studies of fluoxetine hydrochloride in capsule formulations. *Journal of Liquid Chromatography and Related Technologies*.

[12] Raggi M. A., Casamenti G., Mandrioli R., Sabbioni C., Volterra V. (2000). A rapid LC method for the identification and determination of CNS drugs in pharmaceutical formulations. *Journal of Pharmaceutical and Biomedical Analysis*.

[13] Maya M. T., Domingos C. R., Guerreiro M. T., Morais J. A. (2000). Determination of the antidepressant fluoxetine in human plasma by LC with UV detection. *Journal of Pharmaceutical and Biomedical Analysis*.

[14] Lucca A., Gentilini G., Lopez-Silva S., Soldarini A. (2000). Simultaneous determination of human plasma levels of four selective serotonin reuptake inhibitors by high-performance liquid chromatography. *Therapeutic Drug Monitoring*.

[15] Berzas Nevado J. J., Villaseñor Llerena M. J., Contento Salcedo A. M., Aguas Nuevo E. (2000). Determination of fluoxetine, fluvoxamine, and clomipramine in pharmaceutical formulations by capillary gas chromatography. *Journal of Chromatographic Science*.

[16] Lacassie E., Gaulier J.-M., Marquet P., Rabatel J.-F., Lachâtre G. (2000). Methods for the determination of seven selective serotonin reuptake inhibitors and three active metabolites in human serum using high-performance liquid chromatography and gas chromatography. *Journal of Chromatography B: Biomedical Sciences and Applications*.

[17] Berzas Nevado J. J., Contento Salcedo A. M., Villaseñor Llerena M. J., Aguas Nuevo E. (2000). Method development and validation for the simultaneous determination of fluoxetine and fluvoxamine in pharmaceutical preparations by capillary electrophoresis. *Analytica Chimica Acta*.

[18] Buzinkaiová T., Polonský J. (2000). Determination of four selective serotonin reuptake inhibitors by capillary isotachophoresis. *Electrophoresis*.

[19] Titier K., Bouchet S., Péhourcq F., Moore N., Molimard M. (2003). High-performance liquid chromatographic method with diode array detection to identify and quantify atypical antipsychotics and haloperidol in plasma after overdose. *Journal of Chromatography B: Analytical Technologies in the Biomedical and Life Sciences*.

[20] Raggi M. A., Bugamelli F., Sabbioni C., Saracino M. A., Petio C. (2005). HPLC-DAD determination of plasma levels of the antipsychotic risperidone and its main metabolite for toxicological purposes. *Journal of Separation Science*.

[21] Mahatthanatrakul W., Nontaput T., Sriwiriyajan S., Ridtitid W., Wongnawa M. (2008). Bioequivalence study of a generic risperidone (Iperdal®) in healthy Thai male volunteers. *Journal of Science and Technology*.

[22] Avenoso A., Facciolà G., Salemi M., Spina E. (2000). Determination of risperidone and its major metabolite 9-hydroxyrisperidone in human plasma by reversed-phase liquid chromatography with ultraviolet detection. *Journal of Chromatography B: Biomedical Sciences and Applications*.

[23] Saracino M. A., de Palma A., Boncompagni G., Raggi M. A. (2010). Analysis of risperidone and its metabolite in plasma and saliva by LC with coulometric detection and a novel MEPS procedure. *Talanta*.

[24] Locatelli I., Mrhar A., Grabnar I. (2009). Simultaneous determination of risperidone and 9-hydroxyrisperidone enantiomers in human blood plasma by liquid chromatography with electrochemical detection. *Journal of Pharmaceutical and Biomedical Analysis*.

[25] Balant-Gorgia A. E., Gex-Fabry M., Genet C., Balant L. P. (1999). Therapeutic drug monitoring of risperidone using a new, rapid HPLC method: reappraisal of interindividual variability factors. *Therapeutic Drug Monitoring*.

[26] Remmerie B. M. M., Sips L. L. A., De Vries R. (2003). Validated method for the determination of risperidone and 9-hydroxyrisperidone in human plasma by liquid chromatography-tandem mass spectrometry. *Journal of Chromatography B: Analytical Technologies in the Biomedical and Life Sciences*.

[27] Lostia A. M., Mazzarini L., Pacchiarotti I. (2009). Serum levels of risperidone and its metabolite, 9-hydroxyrisperidone: correlation between drug concentration and clinical response. *Therapeutic Drug Monitoring*.

[28] De Meulder M., Remmerie B. M. M., de Vries R. (2008). Validated LC-MS/MS methods for the determination of risperidone and the enantiomers of 9-hydroxyrisperidone in human plasma and urine. *Journal of Chromatography B: Analytical Technologies in the Biomedical and Life Sciences*.

[29] Bhatt J., Subbaiah G., Singh S. (2006). Liquid chromatography/tandem mass spectrometry method for simultaneous determination of risperidone and its active metabolite 9-hydroxyrisperidone in human plasma. *Rapid Communications in Mass Spectrometry*.

[30] Rogóz Z., Skuza G., Daniel W. A., Wójcikowski J., Dudek D., Wróbel A. (2007). Amantadine as an additive treatment in patients suffering from drug-resistant unipolar depression. *Pharmacological Reports*.

[31] Mueller N. (2010). COX-2 inhibitors as antidepressants and antipsychotics: clinical evidence. *Current Opinion in Investigational Drugs*.

[32] Rasmussen K. (2006). Creating more effective antidepressants: clues from the clinic. *Drug Discovery Today*.

[33] Yoshimura R., Umene-Nakano W., Ueda N., Ikenouchi-Sugita A., Hori H., Nakamura J. (2008). Addition of risperidone to sertraline improves sertraline-resistant refractory depression without influencing plasma concentrations of sertraline and desmethylsertraline. *Human Psychopharmacology: Clinical and Experimental*.

[34] Keitner G. I., Garlow S. J., Ryan C. E. (2009). A randomized, placebo-controlled trial of risperidone augmentation for patients with difficult-to-treat unipolar, non-psychotic major depression. *Journal of Psychiatric Research*.

[35] U.S. Department of Health and Human Services, Food and Drug Administration, Guidance for Industry, *Bioanalytical Method Validation*, May 2001

